# Adolescents’ height and cognitive ability in China

**DOI:** 10.1016/j.heliyon.2024.e28742

**Published:** 2024-03-27

**Authors:** Xiao-Hang Guo, Meng-Ying Wang, Li-Chen Chou

**Affiliations:** a Department of Applied Economics, Shantou University, Shantou, China; b School of Modern Business, Zhejiang Gongshang University, Hangzhou, China

**Keywords:** Height, Cognitive ability, Non-cognitive ability, Instrumental variables

## Abstract

Cognitive ability, as an early human capital, has always been an important research object in modern education and labor economics. Despite growing awareness of the importance of height in individual growth and development, there are few empirical studies on height and cognitive ability. Using the data from the China Education Panel Survey, this paper examined the impact of height on the cognitive ability of adolescents and explored the reasons behind the Chinese pursuit of height growth and the potential impact mechanism. In this paper, comprehensive analysis ability was taken as the representative of cognitive ability. The empirical results showed that height was positively correlated with cognitive ability. From the perspective of the influence mechanism, the hypothesis that height reflected self-esteem, health, non-cognitive ability, and other influences on cognitive ability was excluded. To correct the errors that endogenous problems may cause, we used the PSM method and “age at first menstruation " and “age at first wet dream” as instrumental variables to correct them. The results showed that height still affected cognitive ability, with taller people having higher cognitive ability.

## 1 Introduction

In 2020, the prestigious journal The Lancet published a blockbuster paper [1]. Over the past 35 years, the average height of 19-year-old male and female adults in China has increased by 8.1 cm and 6.1 cm, respectively, the first and third fastest among 200 countries and regions, according to the paper, which was based on 65 million samples and 2181 existing studies. Obviously, Chinese people are getting taller and taller, but the obsession among Chinese people for height is getting deeper and deeper. It seems that if people are taller, they will have a bright future and be more competitive, whether in work, study, interpersonal communication, marriage, or love. Indeed, many studies have found a highly positive correlation between height and cognitive ability and labor wages, so Chinese people's obsession with height is not groundless.

With China's per capita wealth continuing to grow rapidly, the height of Chinese people also shows a growing trend. Height is representative of health and nutrition, which affects the development of individuals. Existing studies mainly explore the impact of height on the perspective of health economics and labor economics. Regarding the impact of height on individual health, the findings mainly support the positive correlation between height and health [2,3,4]. As for the impact of height on individual development, the research results mainly support the positive correlation between height and salary, position, etc. [5]. Although the relationship between height and an individual's growth and development is a vital topic widely discussed, most studies only focus on the positive relationship between height and various indicators, as for whether height premium represents only the influence of other external environments or congenital influence or whether height exerts its effect through other factors such as mental health, self-esteem, evolution, and other mechanisms, few kinds of literature have been involved.

As a kind of initial human capital, improving cognitive ability is conducive to the accumulation of human capital on the micro level, and the improvement of its future education level, income level, and social status. And it can enhance society's innovation ability and promote the regional economy's high-quality growth on the macro level. This paper uses data from the China Education Panel Survey (CEPS) to explore height premium. The family background of the adolescents, various variables of mental and physical health, family background, and test performance of cognitive ability were recorded in CEPS. This paper aims to explore whether height has a positive effect on cognitive ability, and if so, through what mechanism? The innovation of this paper is to first list the possible hypotheses according to the literature and then add the proxy variables of these hypotheses into the regression model, in turn, to observe the changes in height coefficient, to deduce the influence of height on cognition and the possible influence mechanism. In addition, few kinds of literature mentioned the endogeneity problem, and almost no one tried to solve the endogeneity problem in the literature. Based on the reference of predecessors, this paper used the PSM method and instrumental variables to solve the endogeneity problem. According to empiric results, height significantly correlates with cognitive ability, which does not affect cognitive ability through various mechanisms.

The remainder of this study is organized as follows: We first review the relevant literature in Section 2 and then propose research hypotheses in Section 3. Section 4 introduces data and selection and measurement of relevant variables, conducts descriptive statistics analysis, and further sets up an econometric model. The estimation results are presented in Section 5. It mainly contains the results of the baseline model, and a discussion of endogeneity (including OLS analysis, PSM analysis, and instrumental variable analysis). In Section 6, mechanism tests are further analyzed. Section 7 provides a brief conclusion.

## 2 Literature review

The conclusions of the studies on height premium all support the positive relationship between height and various developmental variables. Some literature only studies the positive relationship between height and salary or uses height as a health indicator to explore the impact of health on salary. For example, Loh [6] used the Survey data from the National Longitudinal Survey of Youth of the United States and founded that the height increases by 6.9 inches. Kortt and Leigh [5] found that every 10 cm increase in male height would lead to a 3% increase in hourly wage. Using Chinese data, Gao and Smyth [7] also found a positive correlation between height and salary. Yang et al [8] found evidence in support of height premium that tall people expect a significantly higher salary in career development. In particular, regression results suggested stronger effects of height premium on female than on male. McGovern et al [9] summarized that a 1-cm increase in adult stature was associated with a 4% increase in wages for men and a 6% increase for women. Thompson and Portrait [10] used the instrumental variable method to analyze whether height has a causal relationship with labor market outcomes. They found that as Dutch men became taller, so did their ability to choose careers, and height appeared to be associated with better jobs and greater intergenerational occupational mobility. Thomas and Strauss [11] argue that height has a large and significant effect on wages: taller men and women earn more. Zhang et al. (2017) believe there is a height premium phenomenon in China's labor market. That is, height significantly impacts income, and workers with height advantages are more likely to enter white-collar jobs or occupations with higher social prestige. Schick and Steckel [12] used the data of NCDS to find that tall children have high cognitive and non-cognitive abilities, which together explain the higher wages of people with a height advantage. Ross and Ferris [13] found that tallness, particularly among men, is associated with authority, capability, and success. Compared to their shorter counterparts, taller men have advantages in both the hiring process and the earnings potential. Mitra [14] concluded that taller women in managerial or professional occupations receive a wage premium of about 2.5% with a one-inch increment in height. Cawley [15] found that a 3-inch increase in the height of a white woman would increase her salary by 4%.

On the other hand, some researchers focus on the study of the influence of height on physical health, like psychological state. Judge and Cable [16] believe that people with high height tend to have a high sense of self-worth, and thus easy to obtain a high position and income. Deaton and Arora [2] found that tall people have a positive and optimistic attitude and know how to enjoy happiness. Adams [17] believed that height relates to sensation seeking, affinity, and locus of control. Persico et al. [18] found that taller people were likelier to join sports clubs in middle school, so they had more friends and developed interpersonal, teamwork, and leadership skills. These skills contributed to wage growth in adulthood. Bittmann. (2020) using data from the Transnational European Social Survey from 19 countries to examined the relationship between height and the likelihood of being in a leadership position in the workplace. The results showed that, controlling for education and occupational status, every centimeter increase in absolute height increased the likelihood of a woman being in a leadership position by 0.15 percentage points, while there was no effect for men.

Some researchers also believe that the positive correlation between height and salary, position, health, and other variables is probably caused by the omission of some important variables. For example, the study by Spears [19] shows that a suitable growth environment, early nutrition, and health have positive effects on height and cognitive ability. Wang et al [20] suggested that the observed height premium is likely to pick up the impacts of several cognitive/noncognitive skills on earnings confounded in previous studies, such as mental health, risk preference, and personality factors. A false correlation between height and cognition may result if endogeneity is not considered.

In fact, the current literature on the influence of height on cognition can be divided into two categories. One is to directly believe that height affects cognition. For example, in the study of Case and Paxson [21], they still found a significant positive correlation between childhood height and cognitive ability after adding socio-economic variables and the mother's fixed effect, and they put the cognitive ability of 5–11 years old into the model. It turns out that adult height has little impact on wages. The other is to list several hypotheses affecting height and then put the proxy variables of these hypotheses into the regression one by one to observe the effect on the height coefficient. For example, Persico et al. [18] first put the height of different ages into the regression and controlled some essential variables simultaneously. It is found that only the height at the age of 16 impacts the salary. Then, the variables representing self-esteem, intergenerational influence, health status, childhood cognitive ability, and whether to join sports associations are included to observe the impact on the height coefficient individually. It is found that only joining sports associations can significantly reduce the original height premium. Other variables did not affect the height coefficient, so it is concluded that the interpersonal capital accumulated by participating in sports associations is an essential mechanism of the influence of height on development.

Parental resource input also plays an important role in adolescents' height and cognition. Parents may make a compensatory investment in resources for children with poor innate health, or they may make a decision to invest more in children with better learning ability [[22], [23], [24]]. Loughran et al. (2008) found that household parental investment can compensate for whatever disadvantage low birth weight conveys. Thomas [25] had used household survey data from the United States, Brazil, and Ghana to examine the relationship between parental education and child height, an indicator of health and nutritional status. In all three countries, the education of the mother has a bigger effect on her daughter's height; paternal education, in contrast, has a bigger impact on his son's height. There are, apparently, differences in the allocation of household resources depending on the gender of the child and these differences vary with the gender of the parent. Chou et al. [26] applyed data from 2012 CGSS, found that the average education and wage levels of offspring whose fathers have experienced the Cultural Revolution and attained at least primary education are significantly lower than those of other groups in the current labor market. In the past literature, most discussions focused on the impact of parental investment on children's height and cognition, while this article mainly studies the impact of children's own height on their cognitive abilities.

## 3 Hypotheses development

Height may directly have a significant effect on cognitive ability, or it may reflect other underlying traits that affect cognitive ability. Hence, this study summarized the underlying traits influencing cognitive ability into self-esteem, physical health, and non-cognitive ability. First, height is an external visual effect. In fact, it will affect others' evaluation of themselves and then affect self-esteem. Thus, we propose that height is positively correlated with self-esteem, and the higher the comprehensive analysis ability is due to the better self-esteem. Secondly, height is a reflection of a person's health, namely mental and physical health, which affects the ability of comprehensive analysis. In the theory of biological evolution, taller people have more survival advantages. Some studies on height and salary point out that height premiums come from a healthy body, and health is the basis of excellence [5,7]. Therefore, the correlation between health and cognitive ability cannot be ignored. In addition, we speculate that height reflects the level of non-cognitive ability, such as social skills, emotional control, and non-cognitive ability affects cognitive ability [12]. Consequently, this paper will focus on these three potential channels to explore the link between height and cognitive ability.

In the first place, height is conducive to enhancing students' self-esteem, thereby improving cognitive ability. Not only does height make one'sappear competitive, but it also tends to boost one's self-esteem ([27]; Vikesh and Jason, 2022). Improved self-esteem contributes to improved cognitive abilities in adolescents. Therefore, Hypothesis 1 and Hypothesis 2 are formulated as follows:

Hypothesis 1 Height promotes adolescents' cognitive ability.

Hypothesis2 Height promotes adolescents' cognitive ability by improving self-esteem.

Secondly, height is well situated to show physical health, thereby improving cognitive ability. As a good indicator of one's fitness level, height has been associated with better physical health (Rashad, 2008). According to existing literature, an individual's health has a strong association with cognitive abilities Marie-Claude et al., 2016 [28]; Crichton et al., 2016 [29]. Hence, we propose the following hypothesis:

Hypothesis 3 Height promotes adolescents' cognitive ability by facilitating physical health.

Thirdly, height contributes to raising non-cognitive ability, thereby improving cognitive ability. Height is likely to be correlated with non-cognitive traits such as ambition and confidence Persico et al. [18]. Taller children have higher average cognitive and non-cognitive test scores. Together, cognitive and non-cognitive abilities explain the height premium[12]. Thus, we propose that there is another possibility that non-cognitive ability influences the improvement of cognitive ability. Based on the analysis, hypothesis 4 is proposed:

Hypothesis 4 Height promotes adolescents' cognitive ability by facilitating non-cognitive ability.

## 4 Research design

### 4.1 Data source

The data comes from the China Educational Tracking Survey (CEPS), which is a large-scale longitudinal survey project with national representation. The study has been approved by the respondents. The CEPS carried out by the institute of Social Science Survey of Peking University CEPS [30]. It is a pity that although China's education system conducts regular surveys and statistics, the relevant data has not been fully released. Taking this study as an example, the latest junior high school data we can find is the 2014–2015 school year survey. In addition, the data source announcement did not reveal regional details, only macro descriptions from several classes, cities, rural areas, etc. The public data is concentrated on students' comprehensive analysis ability. This survey statistic is primarily designed to measure students' problem-solving abilities rather than general subject achievement tests. According it, in this study we take the 2014–2015 school year as the baseline, the first grade (seventh grade) and the third grade (ninth grade) cohort as the survey starting point, and the average education level of the population and the proportion of the floating population as Stratified variables randomly selected 28 county-level units (counties, districts, cities) from across the country as survey points. The survey was carried out on a school-based basis. 438 classes in 112 schools in 28 county-level units were randomly selected across the country to conduct a questionnaire survey. At the same time, comprehensive cognitive ability tests and basic personality tests were also conducted on students. CEPS tracked and collected micro-data at multiple levels, such as individuals, families, and schools, providing rich data for relevant research and public policy evaluation in education.

### 4.2 Main variables

#### 4.2.1 Cognitive ability

The explained variable used in this paper is a comprehensive analytical ability, measured by scores (0–35). Standardized cognitive ability values are available in the survey database. The student's cognitive ability is measured by a set of approved cognitive ability test questions that do not address specific memorization knowledge taught in the school curriculum but instead measure the student's logical thinking and problem-solving skills. And it has the characteristics of international comparability and national standardization. By standardizing the raw scores obtained from the student's cognitive ability test, we can use the score to measure the comprehensive analytical ability. The higher the score, the stronger the comprehensive analysis ability and the higher the cognitive ability.

#### 4.2.2 Height

The primary explanatory variable was height, with centimeters as the calculation unit. Weight can also be used as an indicator of development, but why do we choose height as an indicator of growth? This is because the change in weight is relatively significant, and the weight may also rise and fall due to factors such as dieting and overeating. Therefore, the selection of height as an indicator of development can eliminate the interference of such factors and obtain relatively reliable results.

#### 4.2.3 Self-esteem

Hypothesis 1 tests the effect of self-esteem on comprehensive analytical ability. Four questions are set in the questionnaire: “The math teacher often praises me”, “the Chinese teacher often praises me”, “the English teacher often praises me”, and “the head teacher often praises me” are assigned 1, 2, 3 and 4 points according to the degree, and then the scores of these four questions are added up. Therefore, self-esteem is a variable ranging from 0 to 16 points. The higher the score, the higher the self-esteem.

#### 4.2.4 Physical health

The variables representing hypothesis 2 are physical health, including physical health and mental health. There are five “self-rated health status” options to measure physical health: “very poor”, “moderately poor”, “average”, “fairly good”, and “very good”. The options given are 5, 4, 3, 2, 1. At the same time, the four conditions of sleep are used to measure physiological health, “whether insomnia”, “whether easy to wake up”, “whether lethargy”, “whether snoring”. The answer of each option is to give 1 point, the answer is no to give 0 points, and then the score of these four questions is added up, the higher the value of the physiological health condition is worse; The mental health questions were divided into nine categories: “Have you been depressed in the past seven days,” “have you been unfocused in the past seven days,” “Have you been unhappy in the past seven days,” “Have you been boring in the past seven days,” “have you been sad in the past seven days,” “have you been nervous in the past seven days,” “have you been worried excessively in the past seven days,” and “have you had a bad feeling in the past seven days. Never, rarely, sometimes, often, and always give 1, 2, 3, 4, and 5 points, respectively, according to the checked options. The score is added up. The higher the score is, the worse the psychological condition is.

#### 4.2.5 Non-cognitive abilities

The variable that represents hypothesis 3, for non-cognitive abilities, is “I sit alone a lot and don't want to be around other people.”, “I don't often talk when I am with my classmates or peers.” These two questions were given 4, 3, 2, and 1 points, for strongly agree, agree, disagree, and strongly disagree. “I can stay calm even when things are bad”, “I am usually confident about the tasks that need to be done”, and the options for these two questions were strongly agree, agree, disagree, and strongly disagree. The options were given 1, 2, 3, and 4 points, respectively, and the score of these four questions was added up to a value ranging from 4 to 16. The higher the score, the worse the non-cognitive ability.

#### 4.2.6 Control variables

This study draws on relevant literature in the field of health and labor economics to control for factors affecting an individual's cognitive ability [[31], [32], [33]]. A close relationship is observed between an individual's cognitive endowment and their family environment. The first part of the control variables is family-related variables. Parents' education level, family income, the number of siblings, and so on may have an impact, so these are included in the model as control variables. In terms of the education level of parents, they can be divided into high school, junior college, bachelor's degree, or above. The regression model takes high school as the control group, and the family's economic status is divided into “very difficult”, “poor”, “ordinary”, “rich” and “very rich”. Regarding the number of siblings in the family, we expected that the more children in the family, the fewer family resources the surveyed students share, which harms students' comprehensive ability. In addition, body weight is also a measure of health capital, so we put it into the model as a control variable, with kilograms as the calculation unit.

### 4.3 Empirical approach

In this paper, height is taken as the primary explanatory variable to estimate the influence of height on students' cognitive ability, namely height premium, and to clarify the mechanism behind the influence: whether height affects adolescents' cognitive ability through three paths, “self-esteem” and “non-cognitive ability” “physical health”, or height directly affects adolescents’ cognitive ability; We can not intuitively understand its transmission mechanism, so we need empirical analysis. Using the human observation unit, we set the ordinary least square model:

(1) $$y_{i}=\beta_{0}+\beta_{1}{∙height}_{i}+\beta_{2}∙{weight}_{i}+\sum_{j=1}^{12}\beta_{3j}∙education+\beta_{4}∙{education}_{i}+{\;\beta }_{5}∙{economic\;situation}_{i}+\sum_{p=1}^{6}\beta_{6p}∙{correlation\;coefficient}_{pi}+\varepsilon_{i.}$$

In equation (1), i stands for each individual, j denotes parents' education level and p is the number of explanatory variables. $y_{i}$ stands for the student i's cognitive ability measured by test scores. ${height}_{i}$ and ${weight}_{i}$ are the student i's height and weight. education is the educational background of the student, and we use dummy variables to measure parents' education. ${education}_{i}$ is the education level of student i. ${economic\;situation}_{i}$ reflects the economic condition of student i's family, for which we use dummy variables to measure family's income. correlationcoefficient is to measure the degree of correlation between explanatory variables and the dependent variable.

## 5 Empirical analysis

### 5.1 Baseline results

Table 1 shows the descriptive statistics of the main variables used in the estimations. The mean value of cognitive ability is approximately 21. In terms of our key independent variable, the average height of male students is higher than that of female students. The average height of male students is 166 cm, and that of female students is 160 cm. The values in the brackets of related variables of each hypothesis are the value range of this variable. The average value of female students is slightly higher than that of male students in respect of self-esteem, mental health, physical health, and non-cognitive ability.

Table 2 presents the main findings. Columns (1) to (3) of Table 2 are samples of all students. Through gradually adding weight, parental education, number of siblings in the family, and family finances, we found that the influence of height on cognitive ability decreased with the addition of these control variables, from 0.065 in model 1 to 0.021 in Column (3). But the results were still significant, representing that the average height increased by 10 cm, and the influence of height on comprehensive analysis ability increased by 0.21. It also means that the effect of height on cognitive ability cannot be explained by adding these control variables, consistent with the expectation of this study. Gender is a dummy variable to distinguish the difference between male and female students in cognitive ability. If the gender is male, it equals one. Otherwise, it equals zero. As shown in column (4) and column (5) of Table 2, based on column (3), we respectively estimated the influence of height on the comprehensive analysis ability of male and female students and found that the influence of height on the comprehensive analysis ability of male students was not significant, while the influence of height on the comprehensive analysis ability of female students was positive and significant. Every 10 cm increase in female height, the influence of height on the comprehensive ability increased by 0.3. In this model, the influence of body weight on the comprehensive analysis ability is significantly negative, which may be because in today's affluent society, heavy body weight, on the contrary, represents poor health, and there may be some diseases or psychological depression due to appearance, which will have a negative impact on the cognitive ability. It is worth mentioning that the influence of weight on the cognitive ability of male is not significant, while the influence of weight on the cognitive ability of female is significant. Like height, it may also indicate that female pay more attention to external beauty than male.

Although it can be seen from Table 2 that height has a positive and significant influence on cognitive ability, we cannot guarantee the accuracy of this result, which is probably caused by other missing variables. That is to say, it may be these missing variables that are the real influencing factors of comprehensive analysis ability, but we have not observed them. Therefore, in this study, we will utilize a couple of strategies to mitigate the endogeneity impact on the estimations by assessing the potential confounding impact of unobservable. In addition, According to Nerlove [34], Wang and Chou [35], if the variance inflation factor (VIF) is greater than 10, the influence of the estimated variable collinearity is higher. The results show that the VIF values of the other variables are less than 10, which means the outcomes avoid the risk of collinearity.

### 5.2 Robustness checks

In the part of OLS, we have tried to include variables that may affect cognitive ability through height in the model. However, it cannot exclude the existence of other missing variables, which may make the initially insignificant variables become significant or bring the possibility of too high or too low. (Look at the literature for examples.) In addition, the measurement error of explanatory variables may also cause the correlation between the residual term and explanatory variables, which is the error of estimation. In the sample of this paper, most of the data were filled in by parents, and the height and weight were not actually measured, so there were inevitable errors among explanatory variables, which would cause endogeneity problems. Therefore, we tried to correct the possible endogeneity problems by using the propensity score method and instrumental variables.

#### 5.2.1 PSM method

The PSM means that, in order to estimate the influence of a major control variable, the propensity score of each sample point is calculated from all other explanatory variables, and then the propensity score is matched according to it. The PSM method can help find the appropriate control group for the selected treatment group and reduce the selection bias. The pairing method mainly includes the neighborhood pairing method, radius pairing method, and Kernel pairing method. In this paper, we take the average height of male and female as the standard, those higher than or equal to the average height is 1, and those less than the average height is 0. We use the dichotomous height variable as the explained variable and use the Logit model to regression estimate the tendency score of other variables. Then the above three matching methods were used to estimate the average effect of height on comprehensive analysis ability. The results are shown in Table 3.

In Table 3, the average height of the tall male is 175 cm, and the average height of the short male is 158 cm. The effects calculated by the three matching methods are all significant, indicating that the comprehensive analysis ability of the tall male is 0.78–0.83 higher than that of the short male. The average height of tall female was 167 cm, and the average height of short male was 154 cm. Among the three matching methods, except the Kernel matching method was not significant, the other two matching methods were significant, and the height effect estimated by PSM was between 0.65 and 0.73.

The estimation method of PSM aims to control the bias of the endogeneity problem on the central core variables through the paired comparison of samples. In the above discussion, we corrected the possible endogenous influence. Still, the estimation method of PSM can only enhance the effect of OLS in the existing control variables that we have mastered and does not actually solve the problem of missing variables. Next, we solve the problem of missing variables by using the variable tool method.

#### 5.2.2 Instrumental variable

Omitted variables may come from students' genes, self-cognition, growth environment, and other unmeasurable factors. This paper exploits the instrumental variable method to alleviate the endogenous problem. Instrumental variables must meet two conditions. One is the correlation of instrumental variables. That is, instrumental variables must have a direct relationship with endogenous variables. Second, the instrumental variables are not correlated with the residual terms of the original model. Teenagers in junior high school are in the period of rapid development. This period is called adolescence. Generally speaking, the stage of puberty begins with the appearance of secondary sex characteristics. However, due to differences between individuals, the time when everyone enters puberty is not consistent, and those who have secondary sex characteristics as early as 10 years old It is precocious puberty. In adolescence, in addition to internal changes in the body such as endocrine changes, the physical appearance of adolescents will also undergo great changes. The most obvious change is the rapid growth of height. Medically, it is generally believed that early puberty occurs in girls between the ages of 10 and 12. Early puberty refers to the obvious characteristics of girls before menstruation, one of which is the rapid increase in height. Middle adolescence is measured from the first menstrual period in girls [36]. Regarding the relationship between girls' height and age at menarche, Rogol's [37] study pointed out that girls who mature earlier are taller in adolescence, but their height in adulthood is not particularly prominent. It can be seen that body size is closely related to the time point of puberty development. Girls who enter puberty earlier may have an earlier height than the time point when menarche occurs. Therefore. We use “age at menarche” as the instrumental variable for height, which should meet the requirements of correlation.

The age of menarche is also consistent with the exogeneity of instrumental variables. Campbell and Udry [38] found that the impact of mother's education on the age of daughter's menarche is much smaller than the mother's own age of menarche. They believed that from a biological perspective, it determines the age of women's menarche. The biggest factor in menstrual age is genes. Kaprio et al. [39] also found that genes are the main factor affecting the age of women's first menstruation in a study using identical and fraternal twins. We used the CEPS sample to conduct regression testing on whether factors such as parents' education level, provincial registration, income, and school location would have an impact on girls' primary education. None of them had significant influence. Here, “age at first menstruation” is used as an instrumental variable, which meets the requirements of exogeneity. In addition, other researchers also have examples of using menstruation as an instrumental variable. For example, Field and Ambrus [40] used the time of women's first menstruation as an instrumental variable to study the association between women's marriage time and education level.

In this paper, we separated male and female samples. The age of the first wet dreams was used as the instrumental variable of height for males, and the age of the first menstruate was used as the instrumental variable of measurement for females. The first stage model of the tool variable is set as follows: $\mu_{i}$ is the residual.

(2) $${height}_{i}=\beta_{0}+\sum_{q=1}^{3}\beta_{qi}∙\text{Age}\;\text{at}\;\text{first}\;\text{menstruation}+\beta_{2}∙{weight}_{i}+\sum_{j=1}^{12}\beta_{3j}∙education+\beta_{4}∙{education}_{i}+{\;\beta }_{5}∙{economic\;situation}_{i}+\sum_{p=1}^{6}\beta_{6p}∙{correlation\;coefficient}_{pi}+\mu_{i}\text{.}$$

The results based on equation (2) are shown in Table 4. In the sample of males, the results of the first stage show that, at the significance level of 1%, the height of male students with wet dreams is 1.7–2.2 cm higher than that of male students without wet dreams. Self-esteem has no significant influence on comprehensive analytical ability, while physical health and non-cognitive ability have a negative and significant influence on comprehensive analytical ability. The height value estimated in the first stage is substituted into the model of the second stage (see equation (3)). The result shows that the model of the second stage is set as follows:

(3) $$y_{i}=\beta_{0}+\beta_{1}{∙height\;estimate}_{i}+\beta_{2}∙{weight}_{i}+\sum_{j=1}^{12}\beta_{3j}∙education+\beta_{4}∙{education}_{i}+{\;\beta }_{5}∙{economic\;situation}_{i}+\sum_{p=1}^{6}\beta_{6p}∙{correlation\;coefficient}_{pi}+\varphi_{i}\text{.}$$

Height still has a significant effect on syn-analytic ability, while self-esteem and physical health have a positive and significant effect on syn-analytic ability. In the female sample, the height of the first stage still has a significant impact on the comprehensive analysis ability. The height of the female with menstruation experience is about 1 cm higher than that of the female without menstruation experience. As for the female with menstruation at the age of 11, there is no significant difference in height, which may be due to the early age of menstruation and fewer samples. Self-esteem has a positive and significant impact on comprehensive analysis ability, while non-cognitive ability has a negative impact on comprehensive analysis ability. The results of the second stage show that mental health has a negative impact on comprehensive analysis ability. We do not know whether it is because female become more sensitive in adolescence and emotional thinking is more remarkable than rational thinking that affects their comprehensive analysis ability. Non-cognitive abilities have a positive effect.

## 6 Mechanism tests

In this section, we followed the method of Persico et al. [18] and gradually added measures related to the above three hypotheses to explore possible mechanisms driving these effects. Table 5 and Table 6, respectively, show the test results of hypothesis 2 to hypothesis 4 for males and females and add self-esteem, physical and mental health, non-cognitive ability, and other variables for analysis. With the addition of self-esteem variables in column (1) of Table 5, Table 6 and it can be seen from the results that for males, self-esteem itself significantly affects comprehensive analysis ability, but height still has no significant influence on comprehensive analysis ability. In the female part, after adding the self-esteem variable, although the self-esteem variable itself has no significant influence on the comprehensive analysis ability, it does not reduce the height premium, and the influence of height on the comprehensive analysis ability is still positive and significant. Therefore, the effect of height premium does not come from the influence of self-esteem on comprehensive analysis ability, so hypothesis 2 is not valid.

Next, in column (2) of Table 5, Table 6, two variables, physical health, and mental health, were added to measure the degree of health. In the male part, the influence of height on comprehensive analysis ability was still not significant; in the female part, the height premium still existed significantly. Although the height coefficient decreased a little, it was still significant at the level of 5%. So, hypothesis 3 does not work. Some researchers believe that height affects non-cognitive ability, such as team leadership ability, and the higher the height, the better the team ability and the better the cognitive ability. In column (3) of Table 5, Table 6, the non-cognitive ability is added to test whether height reflects the level of non-cognitive ability. According to the result analysis, the influence of non-cognitive ability on the comprehensive analytical ability of males and females was not significant; In the male part, the influence of height on comprehensive analysis ability is still not significant, while in the female part, the height premium still exists significantly. Therefore, according to the regression results, hypothesis 4 is not valid.

Column (4) in Table 5, Table 6 puts all the coefficients related to the above hypotheses into the regression form. The influence of height on comprehensive ability remains unchanged. In the male part, the height coefficient does not change, and the influence on comprehensive ability is still not significant. In the female segment, the effect of height on comprehensive analytical ability remained significant at the 5% level. Therefore, it can be shown that hypothesis 1 is always valid, and the influence of height on comprehensive ability is not the result of the influence of hypothesis 2 to hypothesis 4.

## 7 Conclusion

This paper finds that height reflects the level of cognitive ability in adolescents, and the influence of height on cognitive ability remains significant even after controlling for relevant variables. The results of this study suggest that the adolescents' height can reflect the level of cognitive abilities, with taller adolescent generally having higher cognitive abilities. In this research, even with the inclusion of self-esteem, health, and non-cognitive abilities, the impact of height on cognitive abilities did not diminish. Therefore, the three approaches of self-esteem, health, and non-cognitive ability cannot provide a clear explanation for the existence of height premium. This aligns with the findings of Cass and Paxson (2008) that adolescents' height itself is an indicative proxy for cognitive abilities. The research indicates that adolescents' cognitive abilities are influenced by the *pace* of development, with adolescents entering puberty earlier showing higher cognitive abilities. Height serves as a reflection of the timing of entering the developmental period, meaning that individuals who start developing early tend to have higher heights during adolescence, indicating higher cognitive abilities.

Secondly, we considered that the setting of the OLS model might bring about endogeneity problems due to missing variables, so we further tested with the PSM method and instrumental variable method, respectively. The average effect of the three matching methods is similar to that of OLS, which means that the estimated effect of OLS has a certain degree of reliability. In addition, we used the time of first wet dreams and the age of first menstruation as instrumental variables to test the correlation between height and development with male and female samples, respectively. From the regression results of the first stage, the height of the male and the occurrence of the first wet dreams indeed correlated; On average, males with wet dreams were about 1.7–2.2 cm taller than those without. In the female sample, the regression results showed that the height of female students was about 1 cm higher than that of those who had not yet menstruated. Substituting height estimates into the second stage estimates showed that both male and female taller individuals had significantly higher cognitive abilities.

In summary, the research indicates that adolescents' cognitive abilities are influenced by the*pace*of development, with adolescents entering puberty earlier showing higher cognitive abilities. Height serves as a reflection of the timing of entering the developmental period, meaning that individuals who start developing early tend to have higher heights during adolescence, indicating higher cognitive abilities. This study contributes to a better understanding of the relationship between height and cognitive abilities among adolescents during puberty. As for whether height influences future income through cognitive abilities, and whether height affects future outcomes such as income, marital status, etc. The results of this paper suggest that the height of adolescents can reflect the level of cognitive ability, and taller people have higher cognitive ability. The three approaches of self-esteem, health, and non-cognitive ability cannot provide a clear explanation for the existence of height premium.

## Data availability

The datasets generated and analyzed during the current study are available from the corresponding author on reasonable request. These datasets were derived from the following public domain resources: http://ceps.ruc.edu.cn/English/Overview/Overview.htm.

## CRediT authorship contribution statement

**Xiao-Hang Guo:** Writing – original draft, Data curation. **Meng-Ying Wang:** Writing – review & editing, Writing – original draft, Conceptualization. **Li-Chen Chou:** Writing – review & editing, Writing – original draft, Software, Methodology.

## Declaration of competing interest

The authors declare that they have no known competing financial interests or personal relationships that could have appeared to influence the work reported in this paper.

## Figures and Tables

**Table 1:** Descriptive Statistics

	Total			Male			Female		
	Sample	Mean	SD	Sample	Mean	SD	Sample	Mean	SD
Cognitive ability (0–35 scores)	10750	21.072	8.969	4884	22.788	7.034	5866	19.644	10.087
Degree of self-esteem (4–16 scores)	9897	9.420	3.273	4881	9.398	3.388	5016	9.441	3.156
Mental Health (5–25 scores)	9891	11.315	4.406	4880	11.039	4.572	5011	11.584	4.222
Physical Health (1–9 scores)	9896	2.503	1.230	4882	2.434	1.220	5014	2.571	1.236
Non-recognitive ability (1–16 scores)	9889	7.948	2.278	4878	7.911	2.312	5011	7.985	2.244
Height (unit: cm)	9858	163.267	8.027	4863	166.251	8.377	4995	160.362	6.460
Weight (unit: kg)	9731	51.567	11.363	4809	53.878	12.320	4922	49.309	9.832

**Table 2:** Effects of height on cognitive ability

	(1)	(2)	(3)	(4)	(5)
	All	All	All	Male	Female
Height (unit: cm)	0.065***	0.031***	0.021**	0.014	0.030**
	(0.009)	(0.011)	(0.011)	(0.014)	(0.015)
Weight (unit: kg)		−0.017**	−0.021***	−0.003	−0.045***
		(0.007)	(0.007)	(0.010)	(0.010)
Gender	−0.517***	−0.171	−0.176		
	(0.147)	(0.147)	(0.147)		
**Father's Education**					
Senior High School		1.670***	1.519***	1.620***	1.424***
		(0.225)	(0.224)	(0.330)	(0.304)
Specialist		1.216***	0.875***	1.024***	0.710**
		(0.250)	(0.251)	(0.371)	(0.339)
University degree		2.467***	2.063***	1.974***	2.129***
or higher		(0.262)	(0.264)	(0.391)	(0.355)
**Mather's Education**					
Senior High School		0.634**	0.400	0.683*	0.123
		(0.246)	(0.246)	(0.364)	(0.333)
Specialist		1.042***	0.730***	0.792**	0.666*
		(0.254)	(0.25)	(0.374)	(0.347)
University degree		2.067***	1.598***	1.435***	1.714***
or higher		(0.271)	(0.273)	(0.405)	(0.369)
Children			−0.654***	−0.595***	−0.710***
			(0.087)	(0.128)	(0.118)
Economic situation			0.836***	0.858***	0.818***
			(0.118)	(0.163)	(0.172)
Constant	Yes	Yes	Yes	Yes	Yes
Sample	9858	9203	9163	4489	4674
R-square	0.005	0.061	0.072	0.064	0.085
VIF	1.346	1.433	1.544	1.237	1.432

Notes: Columns (1) to (3) use all samples and gradually increase influencing factors to estimate height and cognitive ability. Columns (4) and (5) regress height and cognitive ability by gender. The regression results for the control variables are reported in the table, as are the tables below. ***p < 0.01, **p < 0.05, *p < 0.1. Standard errors in parentheses are clustered at the household level.

**Table 3:** Results in the estimation of propensity score matching

	All			Male			Female		
	(1)	(2)	(3)	(4)	(5)	(6)	(7)	(8)	(9)
	Neighbor Matching	Caliper Matching	Kernel Matching	Neighbor Matching	Caliper Matching	Kernel Matching	Neighbor Matching	Caliper Matching	Kernel Matching
Height_h (=1, higher than average height)	0.251	0.421***	0.415***	0.796**	0.825***	0.777***	0.649**	0.726***	0.220
	(0.242)	(0.189)	(0.183)	(0.396)	(0.315)	(0.303)	(0.313)	(0.245)	(0.313)
Average Height (unit: cm)					166.251			160.362	
Matching sample									
Height_h (=1)	4344	4344	4344	2971	2971	2971	1372	1372	1374
Height_h (=0)	4971	4791	4791	1503	1477	1503	3273	3273	3289

Notes: This table reports the estimation for the findings related to robustness checks of PSM by gender. ***p < 0.01, **p < 0.05, *p < 0.1. Standard errors in parentheses are clustered at the household.

**Table 4:** Robustness check using instrumental variables.

	Male		Female	
	First stage	Second stage	First stage	Second stage
Height		0.231**		0.694**
		(0.113)		(0.300)
Instrument Variable				
Age at first wet dream:				
12	2.205***			
	(0.414)			
13	1.698***			
	(0.287)			
14	1.928***			
	(0.310)			
Age at first menstruation:				
11			0.156	
			(0.387)	
12			0.951***	
			(0.225)	
13			0.951***	
			(0.225)	
Degree of self-esteem	0.049	0.070**	0.068**	−0.029
	(0.032)	(0.032)	(0.028)	(0.042)
Mental Health	0.029	−0.018	0.013	−0.142***
	(0.025)	(0.025)	(0.022)	(0.029)
Physical Health	−0.407***	0.205**	−0.067	0.046
	(0.093)	(0.104)	(0.074)	(0.099)
Non-cognitive ability	−0.103**	−0.033	−0.110***	0.224***
	(0.050)	(0.050)	(0.041)	(0.063)
Control variable	Yes	Yes	Yes	Yes
Constant	Yes	Yes	Yes	Yes
Sample	4480	4480	4298	4298
Weak instrument test	25.481		6.498	
Over identification test (P-value)	1.473		0.144	

Notes: This table shows the results of 2SLS probit regressionby gender. ***p < 0.01, **p < 0.05, *p < 0.1.Standard errors in parentheses are clustered at the household.

**Table 5:** Mechanisms-the effects of self-esteem, physical health, and non-cognitive ability on cognitive ability based on the male sample.

	(1)	(2)	(3)	(4)
Height	0.015	0.015	0.013	0.015
	(0.014)	(0.014)	(0.014)	(0.014)
Degree of self-esteem	0.057*			0.061**
	(0.030)			(0.030)
Mental Health		−0.012		−0.009
		(0.023)		(0.024)
Physical Health		0.106		0.114
		(0.088)		(0.090)
Non-recognitive ability			−0.026	−0.055
			(0.044)	(0.048)
Control variable	Yes	Yes	Yes	Yes
Constant	Yes	Yes	Yes	Yes
Sample	4488	4486	4484	4480
R-square	0.064	0.064	0.064	0.065
VIF	1.301	1.312	1.311	1.504

Notes: ***p < 0.01, **p < 0.05, *p < 0.1. Standard errors in parentheses are clustered at the household.

**Table 6:** Mechanisms-the effects of self-esteem, physical health, and non-cognitive ability on cognitive ability based on the female sample.

	Model 1	Model 2	Model 3	Model 4
Height	0.032**	0.028**	0.030*	0.031**
	(0.016)	(0.016)	(0.016)	(0.015)
Degree of self-esteem	0.019			0.018
	(0.029)			(0.030)
Mental Health		−0.108***		−0.013***
		(0.023)		(0.045)
Physical Health		0.057		0.020
		(0.080)		(0.082)
Non-cognitive ability			0.029	0.113**
			(0.041)	(0.045)
Control variable	Yes	Yes	Yes	Yes
Constant	Yes	Yes	Yes	Yes
Sample	4673	4667	4668	4663
R-square	0.085	0.091	0.085	0.092
VIF	1.279	1.292	1.320	1.497

Notes: ***p < 0.01, **p < 0.05, *p < 0.1. Standard errors in parentheses are clustered at the household.
