# Supplementary material for: Adolescents’ height and cognitive ability in China
Source: Heliyon. 2024 Mar 27;10(7):e28742. doi: 10.1016/j.heliyon.2024.e28742 (PMC10999994; doi:10.1016/j.heliyon.2024.e28742)
Supplement: Multimedia component 1 [file mmc1.pdf]

China Education Panel Survey
Academic Year 2014-2015
Student Questionnaire for Grade 8

Class: __________

Student Name: __________

Name of Homeroom Teacher: __________

Province (Autonomous Region/Municipality): ______________

County (District): ___________ **City: ______________**

Name of School: ____

- -

Students answering this questionnaire do not need to fill in the blanks in the box below.

Code of Questionnaire: [____|____|____|____|____|____]

Code of County/District: [____|____|____|____|____|____]

Hi Dear Student,

China Education Panel Survey is the first large-scale, nationwide, and longitudinal social survey of junior high students in China. It is jointly conducted by the National Survey Research Center (NSRC) at Renmin University of China and academic institutions throughout the country. Last year, we surveyed nearly 20,000 students as well as their parents, homeroom teachers, subject teachers and school administrators; you are one of them and we appreciate your participation. This year, we hope all of you can fill in another questionnaire to help us understand your growth and progress you made during this period.

There are neither right nor wrong answers to these questions and your answers will not be evaluated as part of your academic record. We pledge here that under *the Statistics Law of the People's Republic of China*, we will hold all the information you are going to provide in strict confidence and it will never be given away to any individual or institution. Thank you for your cooperation.

When answering a multiple-choice question, please circle the number of your choice; when answering a blank-filling question, please write down words or numbers on the lines in the square brackets.

National Survey Research Center (NSRC), Renmin University of China

March, 2015

Part A: Personal Background

A1.The location of your Hukou[1] *AT PRESENT* is:

1. In the local county/district
2. Not in the local county/district: County/district: [_________], City: [_________], Province (Autonomous Region/Municipality): [_________]

A2.What is the type of your Hukou *AT PRESENT*? (Agricultural Hukou refers to rural Hukou, a record that identifies a person as a rural resident. Non-agricultural Hukou refers to urban Hukou. Residential Hukou is a general record of residence assigned to all residents of a certain region, regardless of rural or urban background)

1. Agricultural Hukou 2. Non-agricultural Hukou
3. Residential Hukou 4. I have no Hukou

A3.Where is your home *AT PRESENT*? (Home refers to the place you go back to after school every day, or at weekends if you live in a boarding school)

1. In the local county/district 2. Not in the local county/district

A4. What is the level of your command of local dialect?

[1] Hukou refers to a record of household registration system in China that identifies a person as a resident of an area.

1. Don’t understand at all   2. Can understand when listening, but cannot speak it
3. Can speak a little bit   4. Can speak mediocre local dialect
5. Can speak fluently

A5. Are you the only child of your family?
1. Yes, I am. **(SKIP TO A7)**
2. No, I am not.

A6.How many *FULL OR HALF* siblings do you have *AT PRESENT*? (Please fill in 0 if you don't have any sibling.)
[___] elder brother(s)   [___] younger brother(s)
[___] elder sister(s)   [___] younger sister(s)

A7.Which of the following people live in the same household with you *AT PRESENT*? (Please mark all that apply.)
1. Biological father   2. Biological mother
3. Stepfather   4. Stepmother
5. Full or half sibling(s)   6. Grandparent(s) on mother's/father's side
7. Other relative(s)   8. Other non-relative(s)

A8.What’s the *CURRENT* marital status of your biological parents? (Please mark all that apply.)

1. Married → **Do they live together?**
1. Yes, they do.   2. No, they don't.

2. Divorced → **a. When did they divorce?**
In the year of [___|___|___|___]

b. To whom did the court grant your custody?
1. Father   2. Mother
3. Other people (Please specify____________)

c. Have they remarried another person after getting divorced? (Please mark all that apply)
1. Father has remarried.
2. Mother has remarried.
3. Neither of them has remarried.

3. My father has passed away. → **Has your mother remarried ever since?**
1. Yes, she has.   2. No, she hasn't.

4. My mother has passed away. → **Has your father remarried ever since?**
1. Yes, he has.   2. No, he hasn't.

A9.Which one of the following do you think best describes the financial conditions of your family *AT PRESENT*?
1. Very poor   2. Somewhat poor   3. Moderate   4. Somewhat rich   5. Very rich

A10.Do you have a writing desk of your own at home?
1. Yes, I do.   2. No, I don't.

A11.How many books do your family own? (not including textbooks or magazines)

1. Very few 2. Not many 3. Some
4. Quite a few 5. A great number

A12.Do your family own a computer and have an access to the Internet?

0. No, we don't.
1. We have a computer but no access to the Internet.
2. Yes, we have both.

A13.What's the *CURRENT* occupation of your father?

1. Government official/cadre
2. Cadre/Official/Administrator of public institutions, enterprises/corporations
3. Scientist, engineer, university professor or other professionals
4. Doctor, lawyer, high school/primary school teacher
5. Accountant, nurse, computer programmer or other technical staff
6. Ordinary staff or worker (for example: secretary, bank clerk, or librarian)
7. Ordinary staff or worker in business or service field (for example: salesperson, agent, cook, barber, or cosmetologist)
8. Technical worker (for example: driver, electrician, plumber, or mechanist)
9. Ordinary worker (for example: porter, or production line worker)
10. Farmer, herdsman, fisherman
11. Elementary worker (for example: cleaner, guard, housekeeper, or sanitation worker)
12. Self-employed worker
13. Unemployed or laid-off worker
14. Other (Please specify_________)

A14. What's the *CURRENT* occupation of your mother?

1. Government official/cadre
2. Cadre/Official/Administrator of public institutions, enterprises/corporations
3. Scientist, engineer, university professor or other professionals
4. Doctor, lawyer, high school/primary school teacher
5. Accountant, nurse, computer programmer or other technical staff
6. Ordinary staff or worker (for example: secretary, bank clerk, or librarian)
7. Ordinary staff or worker in business or service field (for example: salesperson, agent, cook, barber, or cosmetologist)
8. Technical worker (for example: driver, electrician, plumber, or mechanist)
9. Ordinary worker (for example: porter, or production line worker)
10. Farmer, herdsman, fisherman
11. Elementary worker (for example: cleaner, guard, housekeeper, or sanitation worker)
12. Self-employed worker
13. Unemployed or laid-off worker
14. Other (Please specify_________)

A15.Does your father often get drunk?

1. Yes, he does. 2. No, he doesn't.

A16. Do your parents quarrel a lot?

1. Yes, they do. 2. No, they don't.

A17.Do your parents get along very well?

1. Yes, they do. 2. No, they don't.

A18.How often did your parents check up on your homework *LAST WEEK*?

1. Never 2. One or two days 3. Three or four days 4. Almost everyday

A19.How often did your parents give instruction on your homework *LAST WEEK*?

1. Never 2. One or two days 3. Three or four days 4. Almost everyday

A20.Do your parents care and are they strict with you about the following?

	**They don't care.**	**They do care about it, but are not strict.**	**They are very strict about it.**
Your homework and examination	1	2	3
Your behavior at school	1	2	3
Whom you make friends with	1	2	3
Your dress style	1	2	3
Time you spend on the Internet	1	2	3
Time you spend on watching TV	1	2	3

A21.How often do your parents discuss the following with you?

	**Your father**			**Your mother**		
	**Never**	**Sometimes**	**Often**	**Never**	**Sometimes**	**Often**
Things happened at school	1	2	3	1	2	3
The relationship between you and your friends	1	2	3	1	2	3
The relationship between you and your teachers	1	2	3	1	2	3
Your worries and troubles	1	2	3	1	2	3

A22.How is the general relationship between you and your father?

1. Not close 2.Not too close nor too far 3.Very close

A23.How is the general relationship between you and your mother?

1. Not close 2.Not too close nor too far 3.Very close

A24.How often do you have dinner with your parents?

1. Never 2.Once a year 3.Once every half year
4. Once a month 5.Once a week 6.More than once a week

A25. How often do you visit museums, zoos, science museums, etc. with your parents?

1. Never 2.Once a year 3.Once every half year

4. Once a month 5.Once a week 6.More than once a week

A26.How often do you go out to watch movies, shows, sports games, etc. with your parents?

1. Never 2.Once a year 3.Once every half year
4. Once a month 5.Once a week 6.More than once a week

A27.What is your parents' requirement on your academic record?

1. Being one of the top five of your class
2. Above the average
3. About the average
4. No special requirement

A28.What is the highest level of education your parents expect you to receive?

1. Drop out now
2. Graduate from junior high school
3. Go to technical secondary school or technical school
4. Go to vocational high school
5. Go to senior high school
6. Graduate from junior college
7. Get a bachelor degree
8. Get a Master degree
9. Get a Doctor degree
10. They don’t care

A29.How do you feel about such expectation?

1. Not stressed at all 2. Not very stressed 3. Normal
4. Somewhat stressed 5. Very stressed

A30. What kind of job do your parents *MOST* expect you to do in the future?

1. Government official, staff of public institutions, civil servant
2. Manager or administrator of enterprises/corporations
3. Scientist/engineer/doctor/programmer/pilot/spaceman
4. Teacher/lawyer/accountant/translator
5. Professional designer (such as costume, gardening, or advertisement designer)
6. Artistic performer (including writer/drawer/host/director/screenwriter)
7. Professional athlete
8. Technical worker (including driver/cook/maintenance staff)
9. Soldier/policeman
10. Medium service staff (including stewardess/nurse/barber/cosmetologist), or ordinary office staff
11. Self-employed (such as opening a store)
12. Other (Please specify: __________)
13. They don’t care
14. Not clear

A31.Where do you think your parents *MOST* expect you to live and work in the future?

1. In rural area
2. In towns/counties
3. In small or medium cities
4. In capital cities

5. In Beijing/Shanghai/Guangzhou
6. Abroad
7. They don’t care
8. Not clear

A32.Are your parents confident about your future?

1. Not confident at all
2. Not so confident
3. Somewhat confident
4. Very confident

(Please continue to finish Part B.)

Part B: Academic Development

B1.How many students were there in your class when you were in *GRADE 6*?

[___] students

B2.*AT PRESENT*, is mathematics difficult for you?

1. Very difficult 2. A bit difficult 3. Not very difficult 4. Not difficult at all

B3.*AT PRESENT*, is Chinese difficult for you?

1. Very difficult 2. A bit difficult 3. Not very difficult 4. Not difficult at all

B4.*AT PRESENT*, is English difficult for you?

1. Very difficult 2. A bit difficult 3. Not very difficult 4. Not difficult at all

B5. How much do you agree with each of the following statements about the main subjects?

	**Strongly disagree**	**Somewhat disagree**	**Somewhat agree**	**Strongly agree**
My mathematics teacher always pays attention to me.	1	2	3	4
My Chinese teacher always pays attention to me.	1	2	3	4
My English teacher always pays attention to me.	1	2	3	4
My mathematics teacher always asks me to answer questions in class.	1	2	3	4
My Chinese teacher always asks me to answer questions in class.	1	2	3	4
My English teacher always asks me to answer questions in class.	1	2	3	4
My mathematics teacher always praises me.	1	2	3	4
My Chinese teacher always praises me.	1	2	3	4
My English teacher always praises me.	1	2	3	4

B6. How much do you agree with each of the following statements about your school life?

	**Strongly disagree**	**Somewhat disagree**	**Somewhat agree**	**Strongly agree**
My parents always receive praises on me from my teacher.	1	2	3	4
My parents always receive criticism on me from my teacher.	1	2	3	4
My homeroom teacher always praises me.	1	2	3	4
My homeroom teacher always criticizes me.	1	2	3	4
Most of my classmates are nice to me.	1	2	3	4
My class is in good atmosphere.	1	2	3	4

	Strongly disagree	Somewhat disagree	Somewhat agree	Strongly agree
I often take part in school/class activities.	1	2	3	4
I feel close to people in this school.	1	2	3	4
I feel bored in this school.	1	2	3	4
I hope that I could transfer to another school.	1	2	3	4

B7.How much time *ON AVERAGE EVERYDAY* did you spend on the following extra-curricular activities *FROM MONDAY TO FRIDAY*?

**Doing homework assigned by teacher:**		
1. 0 hour	2. Less than 1 hour	3. About 1-2 hours
4. About 2-3 hours	5. About 3-4 hours	6. More than 4 hours
**Doing homework assigned by parents or cram school:**		
1. 0 hour	2. Less than 1 hour	3. About 1-2 hours
4. About 2-3 hours	5. About 3-4 hours	6. More than 4 hours
**Taking cram school courses (related to schoolwork):**		
1. 0 hour	2. Less than 1 hour	3. About 1-2 hours
4. About 2-3 hours	5. About 3-4 hours	6. More than 4 hours
**Taking interest related courses (not related to schoolwork):**		
1. 0 hour	2. Less than 1 hour	3. About 1-2 hours
4. About 2-3 hours	5. About 3-4 hours	6. More than 4 hours
**Watching TV:**		
1. 0 hour	2. Less than 1 hour	3. About 1-2 hours
4. About 2-3 hours	5. About 3-4 hours	6. More than 4 hours
**Surfing on the Internet or playing video games:**		
1. 0 hour	2. Less than 1 hour	3. About 1-2 hours
4. About 2-3 hours	5. About 3-4 hours	6. More than 4 hours

B8.How much time *ON AVERAGE EVERYDAY* did you spend on the following extra-curricular activities *ON WEEKENDS*?

**Doing homework assigned by teacher:**		
1. 0 hour	2. Less than 2 hour	3. About 2-4 hours
4. About 4-6 hours	5. About 6-8 hours	6. More than 8 hours

**Doing homework assigned by parents or cram school:**		
1. 0 hour	2. Less than 2 hour	3. About 2-4 hours
4. About 4-6 hours	5. About 6-8 hours	6. More than 8 hours
**Taking cram school courses (related to schoolwork):**		
1. 0 hour	2. Less than 2 hour	3. About 2-4 hours
4. About 4-6 hours	5. About 6-8 hours	6. More than 8 hours
**Taking interest related courses (not related to schoolwork):**		
1. 0 hour	2. Less than 2 hour	3. About 2-4 hours
4. About 4-6 hours	5. About 6-8 hours	6. More than 8 hours
**Watching TV:**		
1. 0 hour	2. Less than 2 hour	3. About 2-4 hours
4. About 4-6 hours	5. About 6-8 hours	6. More than 8 hours
**Surfing on the Internet or playing video games:**		
1. 0 hour	2. Less than 2 hour	3. About 2-4 hours
4. About 4-6 hours	5. About 6-8 hours	6. More than 8 hours

B9. How often did you visit museums, zoos, science museums, etc. either alone or with your schoolmates *IN THE PAST YEAR*?

1. Never　2. Once a year　3. Once every half year
4. Once a month　5. More than once a month　6. More than once a week

B10. How often did you go out to watch movies, shows, sports games, etc. either alone or with your schoolmates *IN THE PAST YEAR*?

1. Never　2. Once a year　3. Once every half year
4. Once a month　5. More than once a month　6. More than once a week

B11. What kind of extra-curricular courses do you take *IN THE PAST YEAR*? (Please mark all that apply.)

0. None
1. Mathematical Olympiad
2. Ordinary Mathematics (not including Mathematical Olympiad)
3. Chinese/Chinese Composition Writing
4. English
5. Painting or drawing
6. Calligraphy
7. Music/Musical instrument
8. Dancing
9. Chess
10. Sports

11. Other (Please specify: __________)

B12. What hobbies do you have? (Please mark all that apply.)

0. None
1. Playing musical instruments (Please specify: __________)
2. Vocal practices/Singing/Dancing/Acting
3. Calligraphy
4. Painting or drawing/Animation
5. Chess (Please specify: __________)
6. Sports (Please specify: __________)
7. Other (Please specify: __________)

B13.Besides the hobbies mentioned above (in question B12), what else do you often like to do? (Please mark all that apply.)

0. None
1. Reading books
2. Doing handcrafts
3. Spending time on mobile phones
4. Playing video games
5. Other (Please specify: ________)

B14.What did you usually do in winter and summer vacations *IN THE PAST YEAR*? (Please mark all that apply.)

1. Taking cram school courses (related to schoolwork)
2. Attending summer/winter camps
3. Helping parents out
4. Going back to hometown
5. Staying at home
6. Other (Please specify: __________)

B15. Do you live in school at night *FROM MONDAY TO THURSDAY*?

1. Yes, I do. 2. No, I don't.

B16. How do you usually go to school from home?

1. On foot
2. By bicycle
3. By electric mobile/electric power cart
4. By motorcycle
5. By city bus
6. By intercity bus
7. By private car
8. By train
9. By ship
10. By underground train
11. Other (Please specify: ________)

B17. Using the kind of transportation you chose in question B16, how long does it take from your home to school?

[___|___|___] minutes

B18. What is the highest level of education you expect yourself to receive?

1. Drop out now
2. Graduate from junior high school
3. Go to technical secondary school or technical school
4. Go to vocational high school
5. Go to senior high school
6. Graduate from junior college
7. Get a bachelor degree
8. Get a Master degree
9. Get a Doctor degree
10. I don't care

B19. What kind of job do you *MOST* expect to do in the future?

1. Government official, staff of public institutions, civil servant
2. Manager or administrator of enterprises/corporations
3. Scientist/engineer/doctor/programmer/pilot/spaceman
4. Teacher/lawyer/accountant/translator
5. Professional designer (such as costume, gardening, or advertisement designer)
6. Artistic performer (including writer/drawer/host/director/screenwriter)
7. Professional athlete
8. Technical worker (including driver/cook/maintenance staff)
9. Soldier/policeman
10. Medium service staff (including stewardess/nurse/barber/cosmetologist), or ordinary office staff
11. Self-employed (such as opening a store)
12. Other (Please specify: __________)
13. I don't care

B20.Where do you most expect to live and work when you grow up?

1. In rural area
2. In towns/counties
3. In small or medium cities
4. In capital cities
5. In Beijing/Shanghai/Guangzhou
6. Abroad
7. I don't care

B21.Are you confident in your future?

1. Not confident at all
2. Not so confident
3. Somewhat confident
4. Very confident

(Please continue to finish Part C)

Part C: Physical and Mental Health

C1. Your *CURRENT* height is: [__|__|__] centimeters.

C2. Your *CURRENT* weight is: [__|__] kilograms (Please notice that the unit of the weight is 'kilogram' rather than 'jin')

C3. What do you think of your body shape?
1. Very thin 2. A bit thin 3. Not fat nor thin 4. A bit heavy 5. Very heavy

C4. Which one of the following best describes your general health condition *AT PRESENT*?
1. Very poor 2. Not very good 3. Moderate 4. Good 5. Very good

C5. How often did you get sick *IN THE PAST YEAR*? (such as getting a cold / fever / cough / diarrheal)
1. Never 2. Seldom 3. Often

C6. How many days have you been absent due to sickness *IN THE PAST YEAR*?

[__|__|__] days

C7. *UP TILL NOW,* have you ever had one of the following serious illnesses?

Kidney disease:	1. I don't know<br>2. No, I haven't<br>3. Yes, I had it when I was [__|__] years old and recovered when I was [__|__] years old (Fill in 99 if you haven't recovered yet)
Lung disease:	1. I don't know<br>2. No, I haven't<br>3. Yes, I had it when I was [__|__] years old and recovered when I was [__|__] years old (Fill in 99 if you haven't recovered yet)
Heart disease:	1. I don't know<br>2. No, I haven't<br>3. Yes, I had it when I was [__|__] years old and recovered when I was [__|__] years old (Fill in 99 if you haven't recovered yet)
Brain disease:	1. I don't know<br>2. No, I haven't<br>3. Yes, I had it when I was [__|__] years old and recovered when I was [__|__] years old (Fill in 99 if you haven't recovered yet)
Upper limb fracture:	1. I don't know<br>2. No, I haven't<br>3. Yes, I had it when I was [__|__] years old and recovered when I was [__|__] years old (Fill in 99 if you haven't recovered yet)
Lower limb fracture:	1. I don't know<br>2. No, I haven't<br>3. Yes, I had it when I was [__|__] years old and recovered when I was [__|__] years old (Fill in 99 if you haven't recovered yet)

C8. What was your eyesight according to your *LATEST* health examination at school?

1. I haven't received any health examination at school.
2. According to the result, my left eye is [__]. [__], and right eye is [__]. [__].

C9. Are you short-sighted?

1. Yes, I am.
   The strength of my left eyeglass is [__|__|__] degrees.
   The strength of my right eyeglass is [__|__|__] degrees.
2. Yes, I am. But I am not clear about the strength of my glasses/lens.
3. No, I am not. **(Skip to C11)**

C10. Are you born with myopia?

1. Yes, I am. 2. No, I'm not. 3. Not clear.

C11. Do you have dental caries / tooth decay?

1. Yes, I have [___] decayed tooth/teeth.
2. No, I have not. **(Skip to C13)**

C12. Have you ever had your tooth decay treated (such as filling a tooth)?

1. Yes, I have. 2. No, I haven't.

C13. How often do you do physical exercise?

Usually [___] days a week, [___|___|___] minutes a day.

C14. Your physical ability level:

For boys to answer: Pull-ups: [___|___] times
For girls to answer: Sit-ups: [___|___] times/minute.

C15. How often do you eat food such as fried food, barbecue, puffed food, or Western fast food?

1. Never 2. Seldom 3. Sometimes 4. Often 5. Always

C16. How often do you have sugary drinks (such as milk tea) or sodas (such as Coke)?

1. Never 2. Seldom 3. Sometimes 4. Often 5. Always

C17. Does any of your family members *WHO LIVE WITH YOU* smoke?

1. Yes. [____] of my family members do/does. 2. No, none of them does.

C18. How much time on average do you sleep *EVERY NIGHT*?

[__|__] hours [__|__] minutes

C19. Generally speaking, do you have any of the following sleeping problems? (Please mark all that apply)

0. I have no sleeping problems
1. Insomnia, difficult to fall asleep
2. Sleep fragmentation
3. Drowsiness
4. Remain fatigue after waking up
5. Snoring
6. Grind teeth in sleep
7. Sleepwalking
8. Dreaminess
9. Talk in sleep

C20. Have you ever taken any health education classes when you were in *ELEMENTARY SCHOOL?*

1. Yes, I have. 2. No, I haven't.

C21. Have you ever taken any health education classes when you were in *MIDDLE SCHOOL*?

1. Yes, I have. 2. No, I haven't.

C22. Your pubertal development:

For boys to answer: My first nocturnal emission occurred when I was [__|__] years old. (Fill in 99 if you haven't experienced one yet).

For girls to answer: My first menstrual period occurred when I was [__|__] years old. (Fill in 99 if you haven't experienced one yet).

C23. How do you view your appearance?

1. Very ugly 2. Somewhat ugly 3. Average-looking
4. Good-looking 5. Very good-looking

C24. How much do you agree with each of the following statements about your experiences in *GRADE 7*?

	Strongly disagree	Somewhat disagree	Somewhat agree	Strongly agree
I would try my best to go to school even if I was not feeling very well or I had other reasons to stay at home.	1	2	3	4
I would try my best to finish even the homework I dislike.	1	2	3	4
I would try my best to finish my homework, even if it would take me quite a long time.	1	2	3	4
I would persist in my interests and hobbies.	1	2	3	4

C25. Do you have the feelings below *IN THE LAST SEVEN DAYS*?

	Never	Seldom	Sometimes	Often	Always
Feeling blue	1	2	3	4	5
Too depressed to focus on anything	1	2	3	4	5
Unhappy	1	2	3	4	5
Not enjoying life	1	2	3	4	5
Having no passion to do anything	1	2	3	4	5
Sad, sorrowful	1	2	3	4	5
Nervous	1	2	3	4	5
Excessive worry	1	2	3	4	5

	Never	Seldom	Sometimes	Often	Always
Feeling something bad will happen	1	2	3	4	5
Too energetic to concentrate in class	1	2	3	4	5

C26. How much do you agree with the following statements?

	Strongly disagree	Somewhat disagree	Somewhat agree	Strongly agree
Usually I can recover from the feelings mentioned above very soon by myself.	1	2	3	4
Usually I can recover from the feelings mentioned above very soon with the help of other people.	1	2	3	4
If I have the feelings mentioned above, my teacher would ignore and isolate me.	1	2	3	4
If I have the feelings mentioned above, my teacher would criticize me in public.	1	2	3	4
If I have the feelings mentioned above, my teacher would criticize me privately.	1	2	3	4
If I have the feelings mentioned above, my teacher would try to help me.	1	2	3	4
If I have the feelings mentioned above, my teacher would ask my parents to help me together.	1	2	3	4

(Please continue to finish Part D)

Part D: Social Behaviors and Development

D1. How often did you do the following things *IN THE PAST YEAR?*

	Never	Seldom	Sometimes	Often	Always
Helping elders	1	2	3	4	5
Following orders and lining up	1	2	3	4	5
Being nice and honest	1	2	3	4	5

D2. How often did you do the following things *IN THE PAST YEAR*?

	Never	Seldom	Sometimes	Often	Always
Cursing or saying swearwords	1	2	3	4	5
Quarreling with others	1	2	3	4	5
Having a fight with others	1	2	3	4	5
Bullying the weak	1	2	3	4	5
Having a violent temper	1	2	3	4	5
Unable to concentrate on one thing	1	2	3	4	5
Skipping classes, being absent, or truanting	1	2	3	4	5
Copying homework from others, or cheating in exams	1	2	3	4	5
Smoking, or drinking alcohol	1	2	3	4	5
Going to net bars or video arcade	1	2	3	4	5

D3. How much do you agree with the following statements?

	Strongly disagree	Somewhat disagree	Somewhat agree	Strongly agree
I'm very shy.	1	2	3	4
Usually I would rather sit alone than join others.	1	2	3	4
I seldom talk and mostly listen to them when I'm with my schoolmates or friends.	1	2	3	4
There are some adults I respect and admire.	1	2	3	4
I can chat with adults easily.	1	2	3	4
I would apologize if I hurt others unintentionally.	1	2	3	4
I would try to find other ways to solve problems if my approach to dealing with things is inappropriate.	1	2	3	4
I can stay calm even in bad situations.	1	2	3	4
Usually, I have confidence in my ability to fulfill my task.	1	2	3	4

D4. Adolescents would have some romantic feelings for someone of opposite sex when they are growing up. Have you ever had such feelings for any of your schoolmates or friends of the opposite sex?
1. Yes, I have.   2. No, I haven’t.

D5. Have you ever been in a relationship?
1. Yes, I have or I’m in a relationship now.   2. No, I have never been in a relationship.

D6. Have you ever done the following things with anyone of the opposite sex? (Please mark all that apply)
1. Holding hands   2. Kissing   3. Other more intimate physical contacts   4. None of the above

D7. What's the attitude of most of your peers toward having relationships among junior high students?
1. Antipathy   2.Indifference   3. Admire
4. Jealousy   5. Others (Please specify__________)

D8. What’s the attitude of your teachers and elder members of your family toward having a romance among junior high students?
1. Opposed   2. Not opposed

D9. How many best friends do you have?
[__|__] friends

D10.Please write down names of 5 of your best friends truthfully in the first column of the table below, and then fill in the blanks with number that best describes their conditions. (Please write down as many best friends as you have if there are less than 5.)

**Names of 5 of your best friends**	**Gender**<br>**1. Male**<br>**2. Female**	**Location of Hukou**<br>**1. In the local county/district**<br>**2. Not in the local county/district**	**Does he/she attend the same school with you?**<br>**1. Yes.**<br>**2. No.**	**Is he/she in the same class with you?**<br>**1. Yes.**<br>**2. No.**
1____________	[____]	[____]	[____]	[____]
2____________	[____]	[____]	[____]	[____]
3____________	[____]	[____]	[____]	[____]
4____________	[____]	[____]	[____]	[____]
5____________	[____]	[____]	[____]	[____]

D11. How many of your best friends mentioned above fit in the following descriptions?

	None of them	One or two of them	Most of them
Doing well in academic performance	1	2	3
Studying hard	1	2	3
Expecting to go to college	1	2	3
Skipping classes	1	2	3
Criticized or punished for violating school rules	1	2	3
Always fighting with others	1	2	3
Smoking or drinking alcohol	1	2	3
Always going to net bars or video arcade	1	2	3
Having had or is having a romance	1	2	3
Dropped out of school	1	2	3

D12. Who will be the first one for you to turn to when you want to chat with someone?

1. Schoolmates or good friends 2. Parents 3. A relative of yours
4. Teachers 5. No one

D13. Who will be the first one for you to turn to when you are in trouble?

1. Schoolmates or good friends 2. Parents 3. A relative of yours
4. Teachers 5. No one

D14. Who will be the first one for you to turn to when you need help?

1. Schoolmates or good friends 2. Parents 3. A relative of yours
4. Teachers 5. No one

D15. Do you think your classmates from the local county/district will make friends with those classmates who come from other *URBAN* counties/districts? (Please give your answers based on your assumption if you don't have any classmate from non-local counties/districts.)

1. Yes, they will 2. No, they won’t

D16. Do you think your classmates from the local county/district will make friends with those classmates who come from other *RURAL* counties/districts? (Please give your answers based on your assumption if you don't have any classmate from non-local counties/districts.)

1. Yes, they will 2. No, they won’t

(Please continue to finish Part E)

Part E: Contact Information

We hope to continue the tracking of your growth and improvement in the next year by follow-up surveys. Please leave the contact information of yours and that of your parents, so that we could keep in touch with you (*WE PROMISE THAT WE WILL HOLD ALL THE INFORMATION YOU PROVIDE IN STRICT CONFIDENTCE*):

E1. Your contact information:

Your QQ number: [___|___|___|___|___|___|___|___|___|___]

Your mobile phone number: [___|___|___|___|___|___|___|___|___|___|___]

Your wechat number: ____________________

E2. Your family's telephone number:

Area code: [___|___|___|___] Telephone number: [___|___|___|___|___|___|___|___]

E3. Your father's contact information:

Name: ___________ Mobile phone number: [___|___|___|___|___|___|___|___|___|___|___]

E4. Your mother's contact information:

Name: ___________ Mobile phone number: [___|___|___|___|___|___|___|___|___|___|___]

E5. The contact information of your 3 best friends:

Name	Mobile phone number:	QQ number
1___________	[__|__|__|__|__|__|__|__|__|__|__]	[__|__|__|__|__|__|__|__|__|__]
2___________	[__|__|__|__|__|__|__|__|__|__|__]	[__|__|__|__|__|__|__|__|__|__]
3___________	[__|__|__|__|__|__|__|__|__|__|__]	[__|__|__|__|__|__|__|__|__|__]

This is the end of the questionnaire. Thank you for your engagement and cooperation! We wish you a happy life and a successful future!
